# Spontaneous Cervical Epidural Hematoma with Hemiparesis Mimicking Cerebral Stroke

**DOI:** 10.1155/2014/210146

**Published:** 2014-10-02

**Authors:** Mehmet Tiryaki, Recep Basaran, Serdar Onur Aydin, Mustafa Efendioglu, Ece Balkuv, Naci Balak

**Affiliations:** ^1^Department of Neurosurgery, Dr. Lutfi Kirdar Kartal Education and Research Hospital, Kartal, 34890 Istanbul, Turkey; ^2^Department of Neurology, Istanbul Medeniyet University Goztepe Education and Research Hospital, 34730 Istanbul, Turkey; ^3^Department of Neurosurgery, Istanbul Medeniyet University Goztepe Education and Research Hospital, 34730 Istanbul, Turkey

## Abstract

*Aim.* Spontaneous cervical epidural hematoma (SCEH) is defined as an epidural hematoma that does not have an etiological explanation. The most common site for SCEH is cervicothoracic area. Early diagnosis and treatment are important for prognosis and good results. In this paper, we aimed to present a case who complains of sudden weakness on right extremities imitating cerebral stroke and that neuroimaging reveals spontaneous cervical epidural hematoma. *Case.* A 72-year-old woman was admitted to our hospital with acute neck pain and loss of strength on right extremities. On neurological examination, the patient had right hemiparesis. PT, aPTT, and INR results were 50.5, 42.8, and 4.8, respectively. Cranial MRI was in normal limits. Spinal MRI revealed a lesion that extends from C4 to C7 located on the right side and compatible with epidural hematoma. The patient was operated after normalization of INR values. *Conclusion.* Even though SCEH is a rare condition, it can cause severe morbidity and mortality. Early diagnosis and treatment are quiet important for prognosis. SCEH can easily be mistaken for stroke as with other pathologies and this diagnosis should come to mind especially in patients who have diathesis of bleeding.

## 1. Introduction 

Spontaneous cervical epidural hematoma (SCEH) is defined as an epidural hematoma that does not have a known etiological reason [1]. Although arteriovenous malformations, tumors, trauma, or postoperative complications are blamed for causes of this situation, the most common site of SCEH is cervicothoracic area [2]. Patients usually complain of acute neck pain or interscapular pain. As a result of spinal cord pressure, sensory and motor loss can be seen [1]. SCEH is an important and urgent cause of spinal cord pressure. Its incidence is estimated as 0,1/100000 patients [3]. Male/female ratio is 1,4/1 [4]. Early diagnosis and treatment are essential for a good prognosis.

We aimed to present a patient with right hemiparesis initially mistaken for a cerebrovascular disease. Further tests revealed cervical epidural hemorrhage and the patient has been operated on.

## 2. Case Report

**Figure 1:**
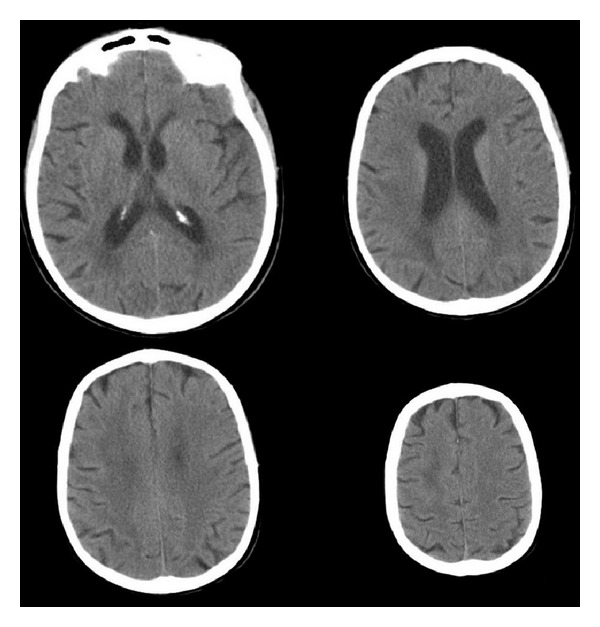
CT scan showed no sign of hemorrhage.

**Figure 2:**
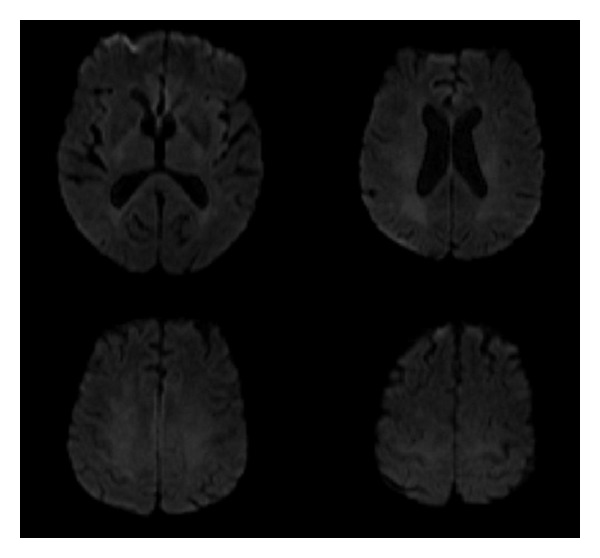
MRI of brain was normal; there is no sign of ischemic or tumor lesion.

**Figure 3:**
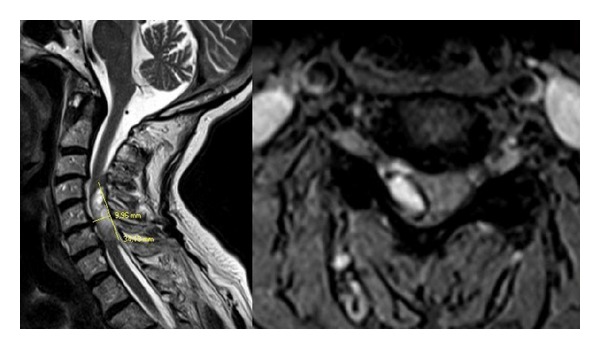
MRI of spine showed epidural hematoma lining right lateral to spinal cord in cervical region.

A 72-year-old female patient was brought to the emergency department with a sudden onset of severe sharp neck pain, upper back pain, and weakness on right arm and leg. Approximately 5 years ago, the patient had a mitral valve replacement and uses warfarin. On neurological examination, her strength was 2-3/5 in right upper limb and 2/5 in right lower limb. In laboratory test results PT value was 50, 5, APTT value was 42, 8, and INR value was 4, 8. Cranial CT scan results were in normal limits and there was no sign of bleeding (Figure 1). Cranial magnetic resonance imaging (MRI) was also within normal limits and there were no evidence pathologies such as tumor or ischemia (Figure 2). Spinal MRI revealed a rightly localized lesion compatible with epidural hematoma that extended from C4 to C7 (Figure 3). Following the infusion of three units of fresh frozen plasma (FFP), INR value was 1.8 and the patient underwent surgery. Right partial C4–C7 and C5-C6 total laminectomy was performed. Thrombosed hematoma has been discharged. On postoperative cervical MRI hematoma was totally emptied. On postoperative neurological examination of the right upper and lower extremity, muscle strength on the right side was 4/5. The patient was able to walk without aid after three months of physical therapy and rehabilitation program.

## 3. Discussion

SCEH was first described by Jackson in 1869 [5]. The first surgery was realized by Bain in 1897 [6]. The annual incidence is estimated as 0,1/100000 [3]. SCEH is defined as the accumulation of blood in the epidural space in the absence of trauma or vertebral iatrogenic interference. Some authors included coagulopathy, vascular malformation bleeding, or hemorrhagic tumor in this recognition; by some authors, only idiopathic bleeding was evaluated in this definition. Idiopathic SCEH constitutes 40–61% of the cases [1]. Most common localization sites are C6 and T12 levels [2].

Lo et al. listed some factors that cause SCEH such as the use of anticoagulants, thrombolytic therapy, uncontrolled hypertension, long-term use of antiplatelet, factor XI deficiency, and congenital disorders such as hemophilia B [7]. There are some controversies whether the source of bleeding is venous or arterial. Many authors claim that the source of bleeding is venous and it is due to a lack of tissue cover in the epidural venous plexus. A sudden pressure increase in the abdominal cavity or thoracic venous pressure is thought to cause tearing and bleeding [8]. After all, according to some authors, because the arterial pressure in the epidural space is higher than the venous pressure and because of the rapid progression of neurological deterioration, the bleeding that causes SCEH is an arterial bleeding not a venous one [1]. In our case, we did not observe any arterial bleeding during surgery. Still, there is a need for more studies in order to clarify the pathogenesis of SCEH.

The most common initial symptom of SCEH is sudden neck or back pain that spreads to a dermatome depending on hematoma's localization area. Due to the compression of the spinal cord and nerve roots, sensation and motor deficits may be seen in the patients. Mostly, paraparesis or quadriparesis is seen depending on the level of compression of the spinal cord. Hemiparesis is a rare clinical feature [9]. Hemiparesis may be produced by anything that interrupts the corticospinal tract from its origin down to the cervical spine. Etiologies include lesions of the cerebral hemisphere as tumor, traumatic brain pathologies, vascular, and infection or lesions of internal capsule, brain stem, and unilateral spinal cord above C5. Hypoglycemia can sometimes be associated with hemiparesis that clears after the administration of glucose [10]. In 2012, Matsumoto et al. reported cases of SCEH that reveal hemiparesis similarly to our case [11]. Unlike cerebral infarction, pain exists in SCEH. Depending on the size of the lesion, the pain may be followed by loss of sensory or motor deficits and motor deficits are seen more frequently [11].

Computed tomography (CT) is the first choice of imaging in the suspicion of cerebral hemorrhage. In the presence of ischemic lesions, diffusion weighted MRI is a better choice for imaging than CT. Cerebral infarction can be detected in diffusion weighted MRI in a couple of hours after the onset of complaints [12]. For the assessment of spinal lesions, MRI gives detailed information about the localization and size of hematomas, spinal cord edema, and severity of the pressure. In the early stages, SCEH is seen iso or hypointense in T1-weighted imaging and hyperintense in T2-weighted imaging [7].

SCEH is usually a surgical emergency. The most effective treatment is to perform a decompressive laminectomy and hematoma evacuation quickly [13]. Conservative treatment may be preferred in patients with no neurological deterioration or cases with serious high surgical risk or regressive complaints. Recently, studies showed that conservative treatment of cervical lesions is associated with poor outcomes [14]. In SCEH, postoperative mortality rate is around 3–6% [8].

The prognosis of SCEH is closely related to the level and size of the hematoma, the degree of preoperative neurological deficit, and the time between the onset of symptoms and surgery. Recent studies showed that hematomas extending between 2 and 10 spinal segments are associated with poor outcomes [15]. Also, surgery performed in the first 36 hours on patients with severe deficits and surgery performed in the first 48 hours on patients with mild deficits increase the possibility of recovery [8]. In a study realized by Shin et al., the surgical recovery rates were found as 83% for the patients operated in the first 12 hours after the onset of initial symptoms, 63.6% for the patients operated between the 12th and the 24th hours after the onset of initial symptoms, and 46.7% for the patients operated 24 hours following the onset of initial symptoms [16].

## 4. Conclusion

Although SCEH is a rare condition, it can cause severe morbidity and mortality. Early diagnosis and treatment are crucial for the best outcomes. SCEH can imitate different pathologies such as a stroke and this diagnosis should come to mind especially in patients with bleeding diathesis.
